# Supplementary material for: Vitamin D Modified DSS-Induced Colitis in Mice via STING Signaling Pathway
Source: Biology (Basel). 2025 Jun 18;14(6):715. doi: 10.3390/biology14060715 (PMC12190092; doi:10.3390/biology14060715)
Supplement: Supplementary file 1 [file biology-14-00715-s001.zip › Supplementary Materials S1: 16s-report of gut microbiota/Groups/diff_analysis/RF/GBM_Groups.RFimportance.pdf]

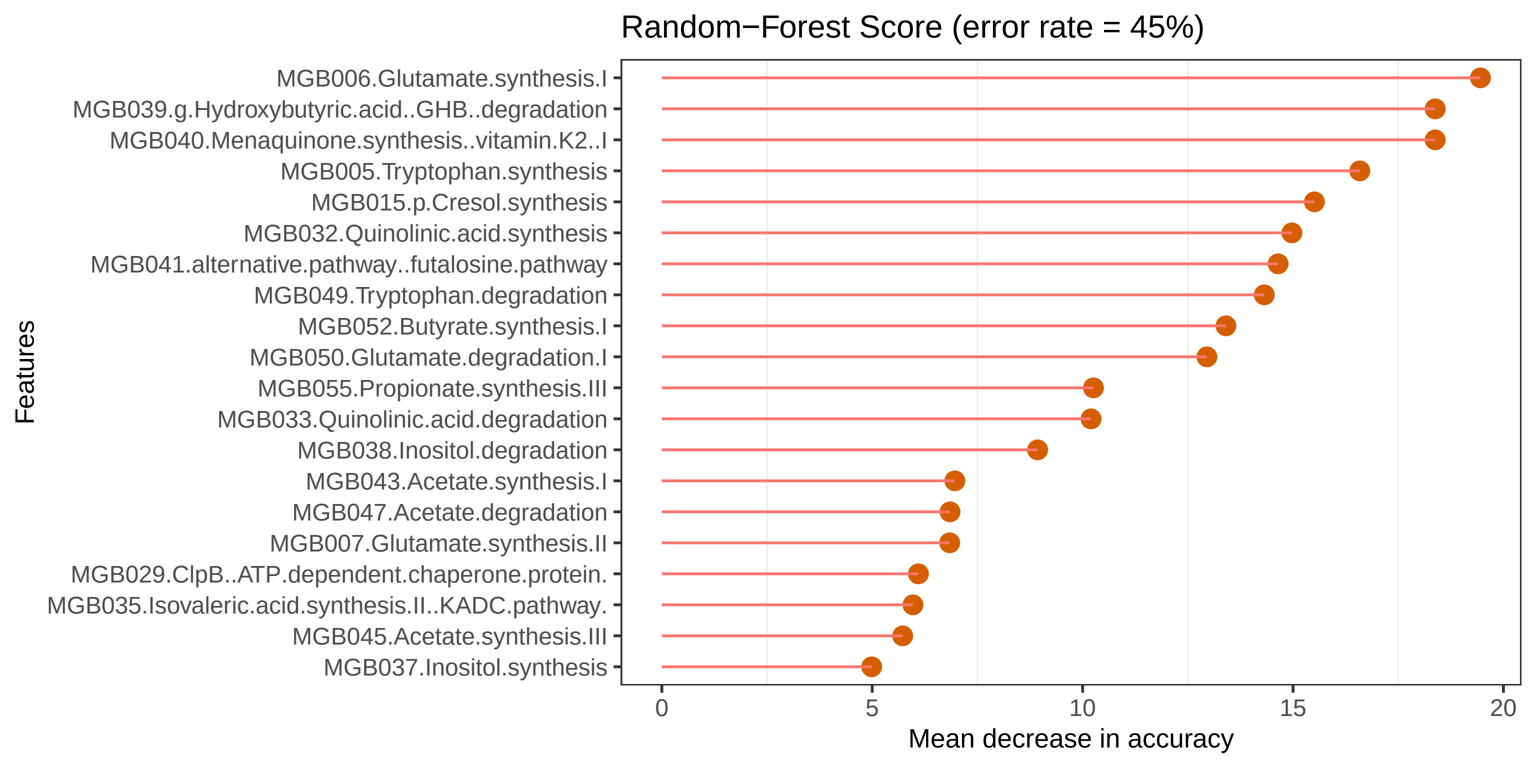

Random−Forest Score (error rate = 45%)
MGB006.Glutamate.synthesis.I
MGB039.g.Hydroxybutyric.acid..GHB..degradation
MGB040.Menaquinone.synthesis..vitamin.K2..I
MGB005.Tryptophan.synthesis
MGB015.p.Cresol.synthesis
MGB032.Quinolinic.acid.synthesis
MGB041.alternative.pathway..futalosine.pathway
MGB049.Tryptophan.degradation
MGB052.Butyrate.synthesis.I
MGB050.Glutamate.degradation.I
MGB055.Propionate.synthesis.III
MGB033.Quinolinic.acid.degradation
MGB038.Inositol.degradation
MGB043.Acetate.synthesis.I
MGB047.Acetate.degradation
MGB007.Glutamate.synthesis.II
MGB029.ClpB..ATP.dependent.chaperone.protein.
MGB035.Isovaleric.acid.synthesis.II..KADC.pathway.
MGB045.Acetate.synthesis.III
MGB037.Inositol.synthesis
Features
0
5
10
15
20
Mean decrease in accuracy
